# Melting properties of peptides and their solubility in water. Part 1: dipeptides based on glycine or alanine†

**DOI:** 10.1039/c9ra05730g

**Published:** 2019-10-14

**Authors:** Hoang Tam Do, Yeong Zen Chua, Jonas Habicht, Marcel Klinksiek, Moritz Hallermann, Dzmitry Zaitsau, Christoph Schick, Christoph Held

**Affiliations:** Laboratory of Thermodynamics, TU Dortmund University Emil-Figge-Str. 70 44227 Dortmund Germany christoph.held@tu-dortmund.de; Institute of Physics, University of Rostock Albert-Einstein-Str. 23-24 18051 Rostock Germany yeong.chua@uni-rostock.de; Competence Centre CALOR, University of Rostock Albert-Einstein-Str. 25 18051 Rostock Germany; Institute of Chemistry, University of Rostock Dr-Lorenz-Weg 2 18051 Rostock Germany; Chemical Institute A. M. Butlerov, Kazan Federal University 18 Kremlyovskaya Street Kazan 420008 Russian Federation

## Abstract

Melting properties (melting temperature, melting enthalpy and heat capacity difference between liquid and solid phase) of biomolecules are indispensable for natural and engineering sciences. The direct determination of these melting properties by using conventional calorimeters for biological compounds is often not possible due to decomposition during slow heating. In the current study this drawback is overcome by using fast scanning calorimetry (FSC) to directly measure the melting properties of five dipeptides (glycyl-glycine, glycyl-l-alanine, l-alanyl-glycine, l-alanyl-l-alanine and cyclo(l-alanyl-glycine)). The experimental melting properties were used as inputs into a thermodynamic solid–liquid equilibrium relation to predict solubility of the dipeptides in water. The required activity coefficients were predicted with PC-SAFT using solubility-independent model parameters. PC-SAFT predicted different solubility profiles (solubility *vs.* temperature) of isomers. The predictions were validated by new experimental solubility data, and the crystal structure of the dipeptides in saturated solution was verified by X-ray diffraction. The different water solubility profiles of isomers (glycyl-l-alanine and l-alanyl-glycine) were found to be caused by the big difference in the melting enthalpy of the two dipeptides. To conclude, combining the PC-SAFT and FSC methods allows for accurate prediction of dipeptide solubility in water in a wide temperature range without the need to fit any model parameters to experimental solubility data.

## Abstract 2

Combination of the PC-SAFT and FSC methods allows for accurate prediction of dipeptide solubility in water in a wide temperature range without the need to fit any model parameters to experimental solubility data.

## Graphical abstract

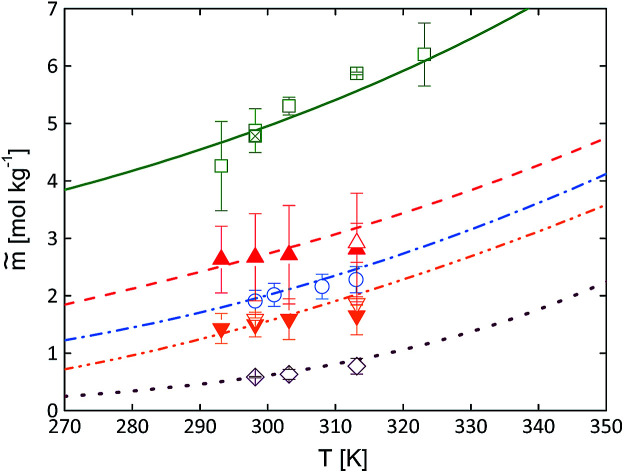

## Introduction

Dipeptides play an essential role in the medicine sector *e.g.* antihypertensive or vasodilatory drugs, sport medicine and tumor therapy.^1^ Furthermore, the dipeptide l-alanyl-l-glutamine is already applied in therapeutic medicine,^2–4^ similar to the dipeptide l-carnosine.^5^ Crystallization is still state-of-the-art unit operation for production and purification of dipeptides. Crystallization requires knowledge about solubility of peptides, while it determines the reaction yield and final purity. Solubility depends on the solvent, and the correct choice of solvent for the crystallization process allows improving the corresponding synthesis and purification processes.^6^ In addition, knowledge is needed in biochemical processes to avoid dipeptide precipitation. The solubility depends on properties of the system such as temperature, nature and concentration of co-solvents and co-solutes and pH value, as well as on the solid phase composition of dipeptides (stability of the crystal phase). Solubility data – also under these influences – can be measured using experimental methods such as photometric and gravimetric methods. However, the experimental determination of solubility is time-consuming and expensive, especially for biological solutions. To circumvent an experimental-based study on solubility, thermodynamic models can be applied that allow predicting solubility behavior given that reliable melting properties are available.

Among such thermodynamic models, *g*^E^ models and equations of state (EoS) are widely used for engineering purposes to calculate the activity coefficients. Models such as the modified Wilson model (Xu *et al.*^7^) with two adjustable parameters per system has already been used to calculate the activity of polymer aqueous solutions as well as the aqueous solubility of several amino acids and dipeptides. Pazuki *et al.* used perturbation theory,^8^ M-Wilson and M-NRTL^9^ models based on three adjustable parameters to predict the activity coefficients of aqueous solutions containing an amino acid or a small peptide. Mortazavi-Manesh *et al.*^10^ used a two-parameter model based on the perturbation theory of a hard-sphere reference to correlate the activity coefficients of some amino acids and peptides in aqueous solutions. Held *et al.*^11^ calculated the activity coefficients based on the Perturbed-Chain Statistical Associating Fluid Theory (PC-SAFT) of aqueous amino-acid and peptide solutions. It has been shown that in comparison to other models, PC-SAFT provides accurate modeling results of activity coefficients and prediction of solubility even in complex mixtures.^12–15^

Thermodynamic models make use of an equilibrium condition between the solid dipeptide and the dipeptide in the saturated liquid phase. No mixed solids (pure compound in single solid phase) were assumed. The temperature dependency of the melting enthalpy was taken in account resulting in the term of the difference of the heat capacities of the solid and liquid state. The difference of heat capacities itself was assumed to be temperature dependent in a linear function. The mole fraction of the dipeptide in the liquid phase at saturation conditions *x*^L,sat^_i_ can be calculated according to ref. 16 and 17 by eqn (1), and by assuming a linear temperature dependence of eqn (2).1
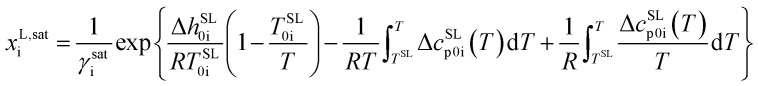
2
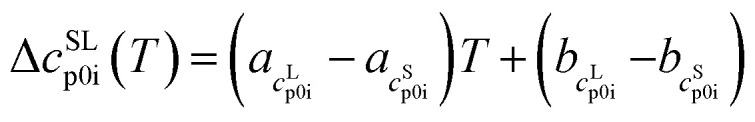
where *γ*^sat^_i_ is the activity coefficient of dipeptide i at the saturated mole fraction *x*^L,sat^_i_ in mole fraction, and *T*^SL^_0i_, Δ*h*^SL^_0i_ and Δ*c*^SL^_p0i_(*T*) are melting temperature, melting enthalpy and heat capacity difference between liquid and solid dipeptide, respectively. Furthermore, Δ*c*^SL^_p0i_ is linear temperature-dependent, with the slopes of the heat capacity 
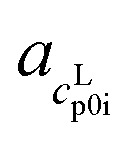
 and 
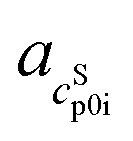
 as well as the interceptions 
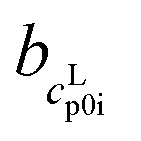
 and 
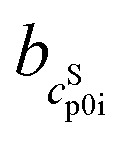
 of the liquid (L) and solid (S) phase. Thus, solubility prediction using eqn (1) require two major contributions: the activity coefficient at saturation and the melting properties (*T*^SL^_0i_, Δ*h*^SL^_0i_ and Δ*c*^SL^_p0i_). The impact of various approximations of the heat capacity difference on the model accuracy has been explored already by Rasmuson's group who promoted a linear function of temperature,^18,19^ while others investigated constant-value approximations.^20,21^

The common strategy to model solubility of amino acids and peptides is to adjust simultaneously all model parameters including melting properties to experimental solubility data in any chosen solvent (in most cases water). This method is physically unsound as so-determined melting properties include information of the mixture. This is forbidden from a physical perspective as melting properties are pure-component properties. As a consequence, so-determined melting properties from literature for several amino acids (Ji and Feng *et al.*^22^ and Ferreira *et al.*^23^) and for dipeptides (Held *et al.*^11^) largely deviate from each other and cannot be considered as reliable data.^24^ The only physically correct procedure to predict solubility is the use of experimentally determined melting properties that are universally valid and do not depend on unphysical treatments. However, the melting properties for amino acids and dipeptides are mostly inaccessible due to the thermal decomposition during slow heating in conventional Differential Scanning Calorimetry (DSC).^25^ However, in our recent work^24^ we have shown that it is possible to use the Fast Scanning Calorimetry (FSC) to avoid thermal decomposition before and during melting. FSC has been successfully applied to accurate study of meting of amino acids glycine and l-alanine,^24^ bio-polymers,^26–28^ low molecular mass compounds^29^ and nucleobases.^30,31^ Further, for glycine and l-alanine we have shown that it is possible to predict the temperature-dependent solubility in water on the basis of the FSC-determined melting properties.^24^

Within this work the temperature-dependent aqueous solubilities of glycyl-glycine (Gly–Gly), glycyl-l-alanine (Gly–Ala), l-alanyl-glycine (Ala–Gly), l-alanyl-l-alanine (Ala–Ala) and cyclo(l-alanyl-glycine) (cyclo(Ala–Gly)) were measured gravimetrically and photometrically. To determine the activity coefficients properly the knowledge about the melting properties are desired according to eqn (1). Due to the decomposition of these dipeptides before melting in common DSC, the melting properties were determined with FSC. The experimental results were used to predict the solubility with PC-SAFT. The predicted solubilities were compared to new experimental solubility data. As cyclization of dipeptides can occur both in solution^32–34^ and in the solid phase^35–37^ during thermal treatment, we have proven that the obtained results corresponded to the dipeptides instead of to their cyclic pendants.

## Methodology

### Materials and reagents

The commercially available dipeptides used in this study are shown Table 1. The dipeptides were used without further purification. Water was directly used from the Millipore-Q-device in the lab.

**Table 1:** Substances, abbreviations, suppliers, CAS numbers and mass-specific purities of the reagents used within this work

Substance	Abbrev.	Supplier	CAS no.	Purity
Glycyl-glycine	Gly–Gly	Sigma A.	556-50-3	≥99%
Glycyl-l-alanine	Gly–Ala	Sigma A.	3695-73-6	≥99%
l-Alanyl-glycine	Ala–Gly	Sigma A.	687-69-4	≥99%
l-Alanyl-l-alanine	Ala–Ala	Bachem	1948-31-8	≥99%
a	Cyclo(Ala–Gly)	Bachem	4526-77-6	≥99%

a Cyclo(alanyl-glycine)((*S*)-3-methyl-2,5-piperazinedione).

Dipeptides might undergo thermally induced cyclization upon heating, as shown in the literatures for l-leucyl-l-leucine^35^ and diphenylalanine.^36^ In order to exclude that cyclization of the dipeptides occurred in our FSC measurements, both Ala–Gly and cyclo(Ala–Gly) were characterized and their melting properties and solubility profiles were compared.

### Measurement of aqueous solubility

The temperature dependence of dipeptides' solubility in water was measured with gravimetric and photometric methods at 100 kPa. The methods are described in detail in the literature.^11,38^ First, Millipore-purified water were filled into Eppendorf® tubes (1.5 mL) and an excessive amount of the studied dipeptide was added to ensure saturation. The compounds in tubes were shaken (850 rpm) for 48 h in a ThermoMixer (Eppendorf) at pre-defined temperature (293.15–323.15 K) with an accuracy of ±0.1 K. Afterwards, the saturated solutions were equilibrated isothermally and without stirring for another 48 h to ensure the solid phase is in equilibrium with the dipeptide in the liquid phase. Four independent saturated solutions were prepared for each dipeptide. At this stage, the pH value of the saturated solutions was measured using glass electrode pH-meter (VWR) with a standard deviation of ±0.03 in pH. For thus prepared saturated solutions with the help of Mettler Toledo XS-205 balance with an accuracy of ±0.01 mg and Specord 210 Plus UV/Vis-spectrometer the solubility values were determined with gravimetric and photometric techniques. In the end the results of the four independently prepared saturated solutions were averaged.

#### Gravimetric method

A sample solution of 100 μL was carefully withdrawn from the saturated liquid phase and weighed. The sample solution was treated in drying chamber at *T* = 298.15 K, *p* = 100 kPa, for more than three days and weighed again. The relative humidity was not considered. To ensure a total evaporation of the solvent, the sample was placed in a vacuum chamber at *T* = 298.15 K and *p* = 2 kPa for 24 h and additionally weighed. The drying procedure was repeated until the evaluated concentration of the dipeptide did not change in the consecutive runs by more than 3%.

#### Photometric method

Calibration curves (absorbance *vs.* molalities) of the dipeptides in undersaturated aqueous solutions were measured in advance in Millipore water. A sample of 3–10 μL was withdrawn from the saturated liquid phase and gravimetrically diluted. The dipeptide-specific dilution factors are shown in Table 2. Each of the diluted solutions was mixed for 1 h and measured spectrophotometrically (Specord 210, Analytik Jena). In all the measurement the final concentration laid within the interval of calibration. In the current work the dipeptides Ala–Gly and Ala–Ala were measured with photometric method. The absorbance maxima are shown in Table 2.

**Table 2:** Dilution factors (DF) and absorbance maximum of the dipeptides measured in this work

Substance	DF	Absorbance maximum
Ala–Gly	14 000	192 nm
Ala–Ala	15 000	190 nm

#### pH correction

The dipeptide solubilities were measured at non-buffered conditions in water. Thus, each of the saturated dipeptide solution has a different pH value. The pH values are listed in Table S4 in the ESI.† In order to compare the dipeptide solubility at same pH conditions, the experimental solubility of each dipeptide was adjusted to pH = 7 using the Henderson–Hasselbalch method (eqn (3) and (4)) for triprotic (Gly–Gly, Gly–Ala, Ala–Gly and Ala–Ala) and diprotic (cyclo(Ala–Gly)) dipeptides according to the following equations:^39^

3 *m̃*^L^_HA,tot_ = *m̃*^L^_HA_[1 + 10^p*K*_a,1_−pH^ + 10^pH−p*K*_a,2_^ + 10^2pH−p*K*_a,2_−p*K*_a,3_^]

4 *m̃*^L^_HA,tot_ = *m̃*^L^_HA_[1 + 10^pH−p*K*_a,1_^ + 10^2pH−p*K*_a,2_−p*K*_a,1_^]

where *m̃*^L^_HA,tot_ represent the total solubility, *m̃*^L^_HA_ the intrinsic solubility of the neutral dipeptide. p*K*_a,1_ p*K*_a,2_ p*K*_a,3_ are the logarithm of acid dissociation constants. These values were calculated using the platform Chemicalize® for the dipeptides considered in this work and listed in Table S1 in the ESI.† The intrinsic solubilities *m̃*^L^_HA_ for all dipeptides at each temperature were determined from eqn (3) or (4) using the pH value of the equilibrated saturated solution and the solubility determined from the saturated unbuffered solution *m̃*^L^_HA,tot_ at the known pH. Based on the calculated intrinsic solubility *m̃*^L^_HA_, the total solubility *m̃*^L^_HA,tot_ at different pH values were calculated. The contribution of the charged and non-charged species of Ala–Ala can be seen in Fig. S2 in the ESI.†

The conversion from the molar fraction *x* [mol mol^−1^] to molality *m̃* [mol kg_water_^−1^] is done according to the following equation:5
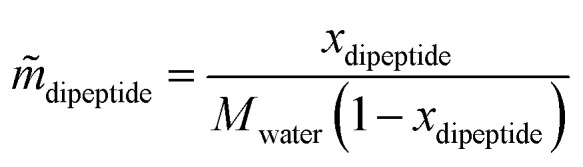
where *x*_dipeptide_ represents the equilibrium mole fraction in [mol mol^−1^], *M*_water_ = 0.018015 kg mol^−1^ the molecular mass of water and *m̃*_dipeptide_ the dipeptide solubility in [mol kg^−1^]. From now the dipeptide solubility will be expressed as molality *m̃*.

#### Powder X-ray diffraction (PXRD)

The crystal structure of the dipeptide that is in equilibrium with its saturated aqueous solution is an important factor for modeling the solubility using eqn (1). It is required that the determined melting properties are measured for the same solid form as the solid precipitate that is in equilibrium with the solute in saturated liquid phase. However, during the dissolving process the crystal structure of the pure dipeptide might change according to the solvent conditions. For example, glycine tends to form different crystal structures in equilibrium with saturated water and in alcohol solutions.^40^ Therefore, the crystal structure of the pure dipeptide without any further treatments was determined at first using PXRD. Then the crystal structure of the solid phase that was in equilibrium with its saturated solution at *T* = 298.15 K was determined.

### Measurement of melting properties

The dipeptides Gly–Gly, Gly–Ala, Ala–Gly and Ala–Ala, as well as cyclo(Ala–Gly) were measured with Mettler Toledo Flash DSC1, which is a Fast Scanning Calorimeter (FSC) equipped with thin film chip sensor USF1,^41^ to determine the melting properties experimentally. The detailed description of the experimental method has been given previously.^24,27,28,30^

**Fig. 1:**
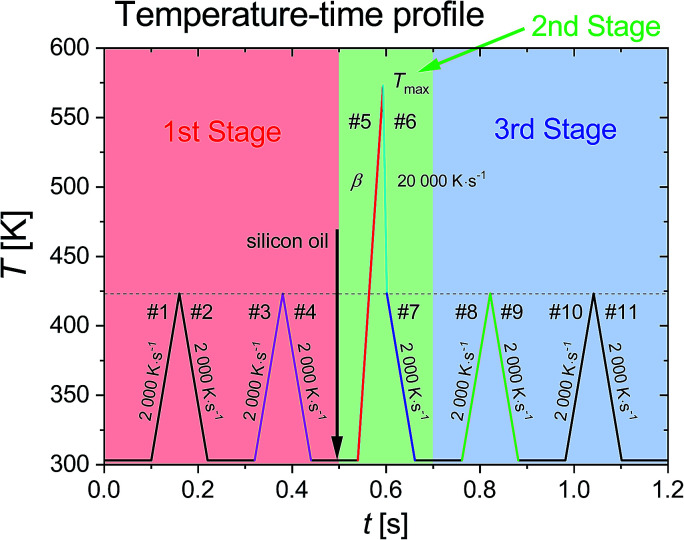
Temperature–time profile with three measurement stages: (i) 1st stage: sample mass determination (red segment), (ii) 2nd stage: sample melting and fast-quenching (green segment), and (iii) 3rd stage: re-heating of supercooled sample (blue segment). After cooling step #4 in 1st stage, the sample can be coated with silicon oil. In the heating step #5, the scanning rate, *β*, varied from 2000 K s^−1^ to 20 000 K s^−1^ [reprinted from ref. 24 with modifications].

The measurement procedure is divided into three measurement stages, as shown in the temperature–time profile in Fig. 1. In the first stage, the initial mass of the sample was determined as *m*_0_ = *C*^S^_p0i_/*c*^S^_p0i_, where *C*^S^_p0i_ is heat capacity of the solid sample on the sensor [J K^−1^] obtained from the heating and cooling cycles in scanning steps #1 to #4, and *c*^S^_p0i_ is specific heat capacity [J g^−1^ K^−1^] obtained from the measurement with conventional DSC (Pyris 1, PerkinElmer, USA). The temperature range and constant scanning rate, *β* = 2000 K s^−1^, were selected as such that the sample undergoes no mass loss, *e.g.* no sublimation and no decomposition.^24,27,28,30^ The starting temperature was set to 303 K.

The properties *T*^SL^_0i_ and Δ*h*^SL^_0i_ are determined in the heating step #5 in the second stage. The Δ*h*^SL^_0i_ is defined as6
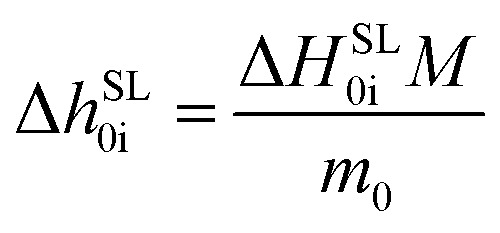
where Δ*H*^SL^_0i_ is the enthalpy, determined as the area under the melting peak in the heat flow curve of heating step #5, *m*_0_ is the sample mass, and *M* is the molar mass of the dipeptide.

**Fig. 2:**
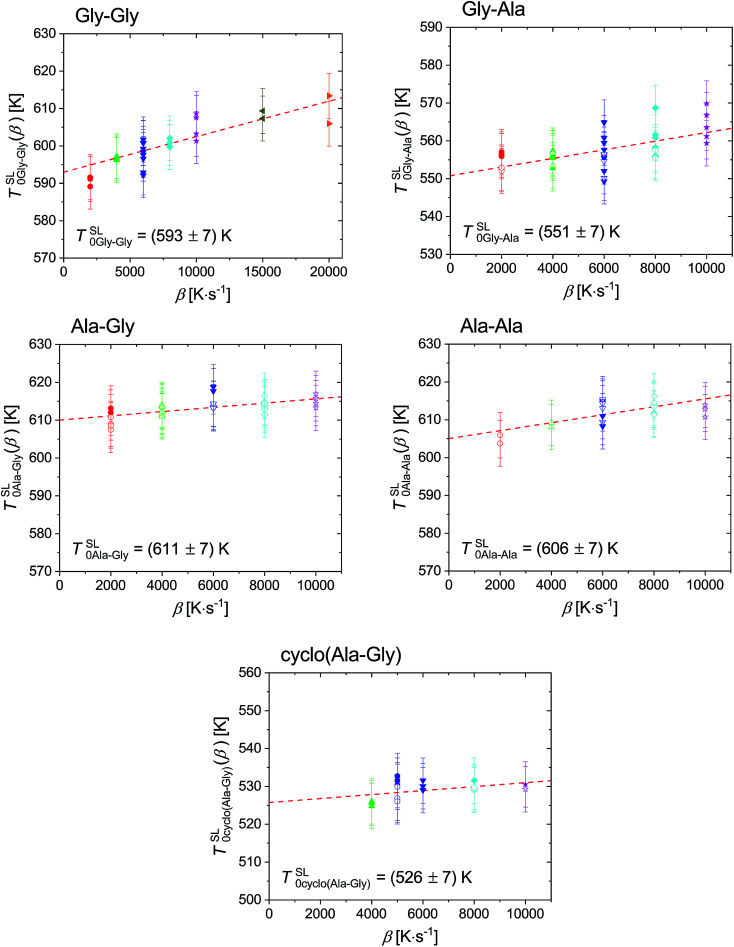
Extrapolated onset temperature of the melting peak of Gly–Gly, Gly–Ala, Ala–Gly, Ala–Ala and cyclo(Ala–Gly), as function of heating rate. The melting temperature at zero heating rate for Gly–Gly, Gly–Ala, Ala–Gly, Ala–Ala and cyclo(Ala–Gly) is *T*^SL^_0Gly–Gly_ = (593 ± 7) K, *T*^SL^_0Gly–Ala_ = (551 ± 7) K, *T*^SL^_0Ala–Gly_ = (611 ± 7) K, *T*^SL^_0Ala–Ala_ = (606 ± 7) K and *T*^SL^_0cyclo(Ala–Gly)_ = (526 ± 7) K, respectively. The scanning rates used were 2000 K s^−1^ (circles), 4000 K s^−1^ (up-triangles), 5000 K s^−1^ (hexagonals), 6000 K s^−1^ (down-triangles), 8000 K s^−1^ (diamonds), 10 000 K s^−1^ (stars), 15 000 K s^−1^ (left-triangles) and 20 000 K s^−1^ (right-triangles). Solid symbols represent sample measurement without silicon oil, while empty symbols for sample measurement with silicon oil.

**Fig. 3:**
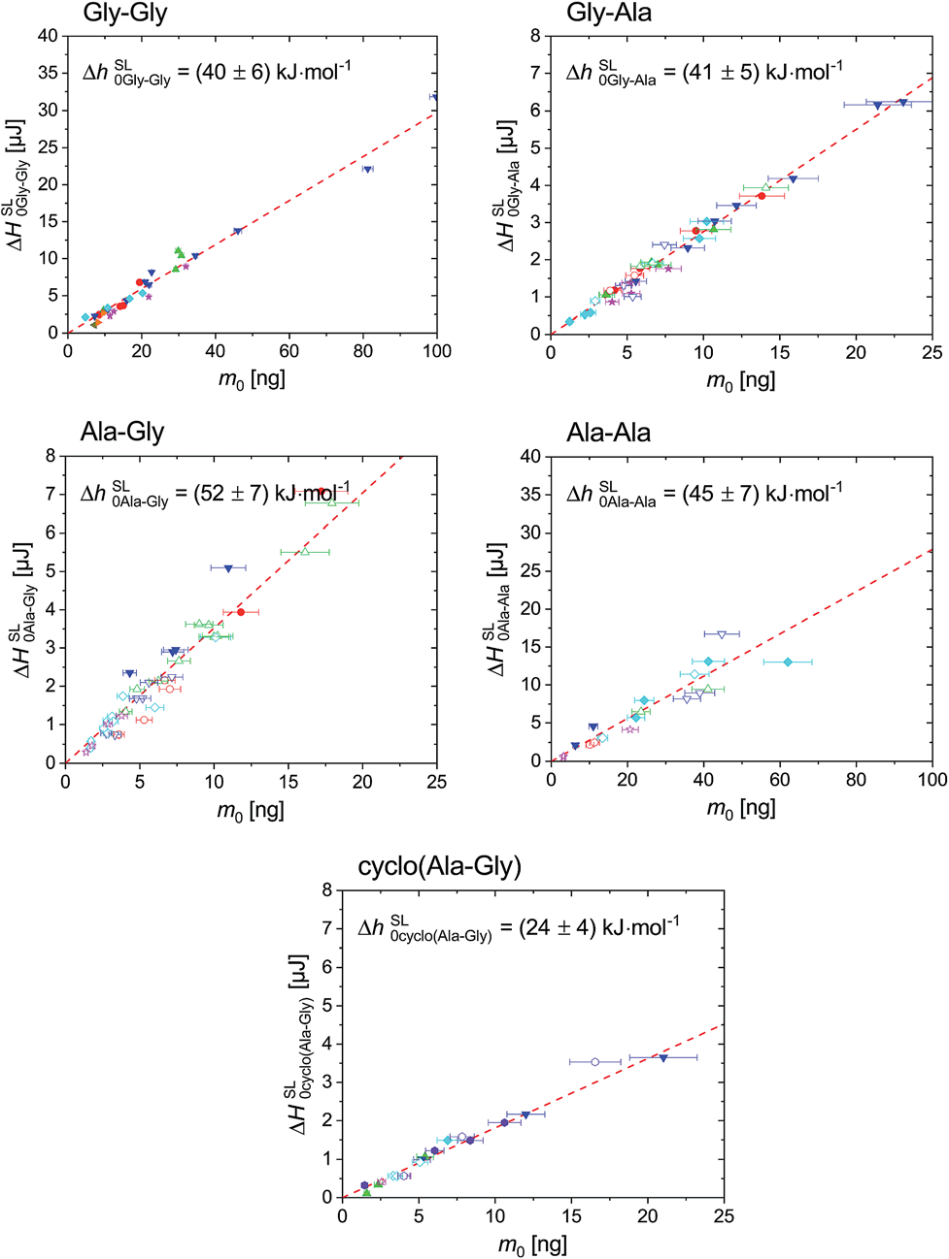
Enthalpy, Δ*H*^SL^_0i_, of Gly–Gly, Gly–Ala, Ala–Gly, Ala–Ala and cyclo(Ala–Gly) in respect to initial sample mass, *m*_0_, regardless of the scanning rates. The symbols used were as described in Fig. 2. The dashed line was linear fit through zero origin, where the slope denoted as Δ*h*^SL^_0i_. The scanning rates used were 2000 K s^−1^ (circles), 4000 K s^−1^ (up-triangles), 5000 K s^−1^ (hexagonals), 6000 K s^−1^ (down-triangles), 8000 K s^−1^ (diamonds), 10 000 K s^−1^ (stars). The melting enthalpy for Gly–Gly, Gly–Ala, Ala–Gly, Ala–Ala and cyclo(Ala–Gly) is Δ*h*^SL^_0Gly–Gly_ = (40 ± 6) kJ mol^−1^, Δ*h*^SL^_0Gly–Ala_ = (41 ± 5) kJ mol^−1^, Δ*h*^SL^_0Ala–Gly_ = (52 ± 7) kJ mol^−1^, Δ*h*^SL^_0Ala–Ala_ = (45 ± 7) kJ mol^−1^ and Δ*h*^SL^_0cyclo(Ala–Gly)_ = (24 ± 4) kJ mol^−1^, respectively. The values are already given in the figures, same for Fig. 2.

In order to ensure good thermal contact between sample and surface of sensor, as well as to decrease the mass loss due to sublimation and evaporation, the sample was coated with silicon oil before the heating step #5. The scanning rate of heating step #5 was varied from 2000 K s^−1^ to 20 000 K s^−1^ used for the extrapolation of the measured properties to zero heating rate. Silicon oil was commonly used to improve the thermal contact in FSC measurements, *e.g.* for polymers^42–45^ and for organic compounds.^24,46^ Nevertheless in order to ensure that there is no interaction between the dipeptides and silicon oil, the melting properties, *T*^SL^_0i_ and Δ*h*^SL^_0i_, for samples coated with and without silicon oil were determined and presented in Fig. 2 and 3.

After melting in heating step #5, the sample without silicon oil was cooled rapidly with a programmed rate of 20 000 K s^−1^ (cooling step #6 in third stage) to minimize the sample mass loss due to evaporation at high temperature. If crystallization in cooling steps #6 and #7 took place, the ultra-fast quenching of the melted sample was applied allowing the sample to retain in the liquid state below the melting temperature (supercooled liquid). The glass transition of the sample (supercooled liquid to glass and *vice versa*) was denoted as a step change in the specific heat capacity.

In the third stage for sample without silicon oil, the glassy sample was heated/cooled in temperature range similar to that in the first stage. The initial scanning rate used in this stage was 2000 K s^−1^, however accessible temperature range for accurate determination of the glass transition with this scanning rate was too narrow. The limitation on the accessible temperature arises due to device response depending on the scanning rate. The accessible temperature range can be increased by (i) decreasing starting temperature below 303 K, and (ii) decreasing the scanning rates of heating and cooling cycles. The first solution is not favorable, as this would increase the measuring time due to increasing required equilibrating time of the device at low temperatures below 303 K. Thus, as in second solution, a range of lower scanning rates was used in heating and cooling cycles in the third stage. By decreasing the scanning rates, the temperature range needed to achieve constant scanning rates decreases. Therefore the accessible temperature range in heating and cooling curves increases. An example of heating and cooling cycles for glassy Gly–Gly in the third stage that shows that the accessible temperature range for glass transition evaluation increases with decreasing scanning rate are shown in Fig. S1 in ESI.†

### PC-SAFT

Modeling solubility using eqn (1) requires the dipeptide activity coefficient. In this work, dipeptide activity coefficients were calculated based on a ratio of fugacity coefficients *φ*_i_/*φ*_0i_ which describes the deviations from the dipeptide at saturated liquid to the pure dipeptide. Fugacity coefficients were calculated by eqn (7)7
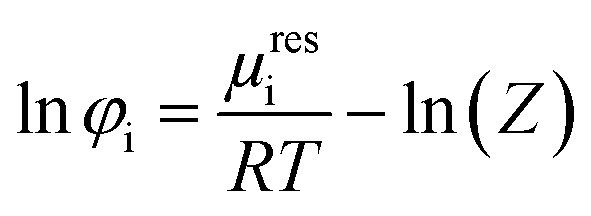
where *μ*^res^_i_ represents the residual chemical potential and *Z* the compressibility factor. In this work, all values were calculated for liquid states. Both, *μ*^res^_i_ and *Z* requires an expression for the residual Helmholtz energy *a*^res^. In this work PC-SAFT was used to calculate the contributions to *a*^res^ according to eqn (8)

8 *a*^res^ = *a*^hc^ + *a*^dip^ + *a*^assoc^

where *a*^hc^, *a*^disp^ and *a*^assoc^ express the Helmholtz-energy contributions of the hard chain repulsion, dispersion and association interactions, respectively. All these contributions have been already published by Gross and Sadowski.^47^ The conventional Berthelot–Lorenz − combining rules were used for interactions between two components i and j (water and dipeptide) in a mixture:9
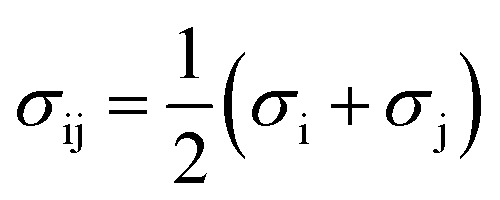
10
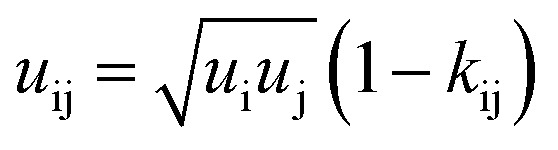


The binary interaction parameter *k*_ij_ is a fit parameter that describes deviations from the geometric mean of the dispersion–energy parameters of components i and j.

The dipeptides used in this work were modeled with the 2B association scheme.^48^ Both, the amino group and the carboxylic group were characterized with one association site each. The PC-SAFT pure-component parameters for the dipeptides were taken from literature.^11^ The parameters were fitted to thermodynamic properties of aqueous solution, and, therefore, depend on the chosen water parameters. Water was modeled as well with the 2B association scheme with a temperature-dependent segment diameter introduced by Cameretti and Sadowski.^49^ The same water parameters used in ref. 24 were also used in the present work. The PC-SAFT parameters used in this work are listed in Table 3.

**Table 3:** PC-SAFT parameters for Gly–Gly, Gly–Ala, Ala–Gly, Ala–Ala, cyclo(Ala–Gly), and water used within this work and *k*_ij_ between dipeptide and water. For all components, the 2B association scheme was applied. The parameters were already published in literature^11^

Component	*m* ^seg^_i_ [−]	*σ*_i_ [Å]	*u*_i_/*k*_B_ [K]	*ε* ^AiBi^/*k*_B_ [K]	*κ* ^AiBi^ [−]	*k*_ij_ to water
Gly–Gly^11^	7.3374	2.327	216.96	2598.06	0.0393	−0.080
Gly–Ala^11^	9.2047	2.411	279.32	2912.21	0.0392	−0.075
Ala–Gly^11^	9.2047	2.411	279.32	2912.21	0.0392	−0.075
Ala–Ala^11^	10.230	2.522	287.59	3176.59	0.0819	−0.074
Cyclo(Ala–Gly)^this work^	5.8185	2.780	278.48	1029.07	0.0157	−0.053
water^49^	1.2047	a	353.94	2425.67	0.0451	—

a For water, a temperature-dependent segment diameter *σ* = 2.7927 + 10.11 exp(−0.01775*T*) − 1.417 exp(−0.01146*T*) was used.

One binary interaction parameter was applied between dipeptide and water according to eqn (10). In this work, the values for *k*_ij_ were fitted to activity-coefficient data of dipeptides in water at (298.15 ± 0.1) K. For cyclo(Ala–Gly) osmotic coefficients in water solutions at (273.15 ± 0.1) K were determined. The Gibbs–Duhem equation was used to convert these values in activity coefficients where the value for *k*_ij_ was fitted to. For comparison, the PC-SAFT modelled and experimental activity coefficients are illustrated in Fig. S3 in the ESI.† The values are listed in Table S2 in the ESI.† Furthermore, the experimental dipeptide-water density data was used to confirm the binary interaction parameter *k*_ij_ values. The result of the parameter fit can be observed in Fig. S4† and the values are listed in Table S3 in the ESI.† It can be seen that PC-SAFT and experimental results are in good agreement. Additionally, it can be observed that the segment number of cyclo(Ala–Gly) as well as the association energy and volume is smaller in comparison with linear Ala–Gly. The molecule Ala–Gly consists of one primary amine group, of one carboxyl group, and of the peptide bond that contains a secondary amine group and a carbonyl group. The molecule cyclo(Ala–Gly) has two peptide bonds, so two secondary amine groups and two carbonyl groups. Thus, the association behavior of cyclo(Ala–Gly) is weaker than of Ala–Gly, which can be seen also from the association parameters listed in Table 3.

## Results and discussion

### Melting temperature and melting enthalpy of dipeptides

The melting properties of dipeptides Gly–Gly, Gly–Ala, Ala–Gly and Ala–Ala, as well as cyclo(Ala–Gly) characterized with fast scanning calorimetry are presented here. The heat flow curves in heating step #5 were used for the determination of the apparent melting temperature, *T*^SL^_0i_ (*β*) and the enthalpy, Δ*h*^SL^_0i_, are shown in Fig. S5 in the ESI,† where the *T*^SL^_0i_ (*β*) was obtained as the onset of the melting peak – the intersect between extrapolated melting peak baseline and auxiliary line through the ascending melting peak slope, while Δ*H*^SL^_0i_ was the area between the melting peak and the baseline.

The extrapolated onset temperatures, *T*^SL^_0i_(*β*), were plotted in respect to heating rates, *β*, as shown in Fig. 2. The value for the thermodynamic melting temperature, *T*^SL^_0i_, is defined as *T*^SL^_0i_ = *T*^SL^_0i_(*β* → 0),^50^ which considers such device dependent effects as the thermal lag^50,51^ and possible superheating.^51–53^ As described above in eqn (6) Δ*H*^SL^_0i_ should linearly depend on the sample mass *m*_0_, regardless of the scanning rate and this was demonstrated for both samples with and without silicon oil in Fig. 3. This also indicates no interaction between dipeptides and silicon oil. The slopes of the dashed red lines in Fig. 3 provides the specific melting enthalpies.^24,27,28^

### Heat capacity change on devitrification of the dipeptides

In the third stage, only ultra-fast quenched melted dipeptides without silicon oil were heated/cooled in the temperature range similar to the first stage with scanning rates from 500 K s^−1^ to 2000 K s^−1^. If crystallization is avoided, a glass transition step from glassy to supercooled liquid state can be observed in heating #8 and cooling #9 steps.

**Fig. 4:**
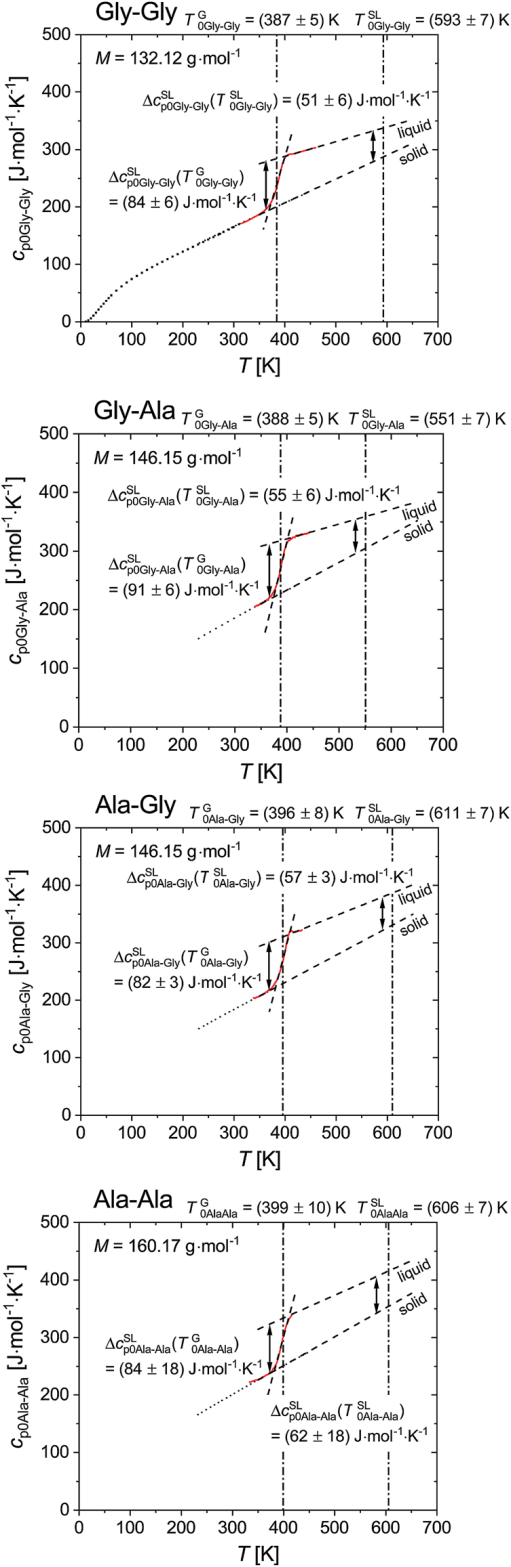
Specific heat capacity for Gly–Gly, Gly–Ala, Ala–Gly and Ala–Ala. The heat capacity of solid, *c*^S^_p0i_, of the dipeptides was measured with conventional DSC (dotted lines). The solid line denotes the glass transition step of ultra-fast quenched melted dipeptides without silicon oil. Both *c*^S^_p0i_ and *c*^L^_p0i_ (dashed lines) were linearly fitted to extrapolate to *T*^SL^_0i_. The heat capacity difference between liquid and solid phase were determined at glass transition temperature, Δ*c*^SL^_p0i_ (*T*^G^_0i_) and at melting temperature, Δ*c*^SL^_p0i_ (*T*^SL^_0i_). Gly–Gly: Δ*c*^SL^_p0Gly–Gly_ (*T*^G^_0Gly–Gly_) = (84 ± 6) J mol^−1^ K^−1^ and Δ*c*^SL^_p_ (*T*^SL^_0Gly–Gly_) = (51 ± 6) J mol^−1^ K^−1^; Gly–Ala: Δ*c*^SL^_p0Gly–Ala_ (*T*^G^_0Gly–Ala_) = (91 ± 6) J mol^−1^ K^−1^ and Δ*c*^SL^_p0Gly–Ala_(*T*^SL^_0Gly–Ala_) = (55 ± 6) J mol^−1^ K^−1^; Ala–Gly: Δ*c*^SL^_p0Ala–Gly_ (*T*^G^_0Ala–Gly_) = (82 ± 3) J mol^−1^ K^−1^ and Δ*c*^SL^_p_ (*T*^SL^_0Ala–Gly_) = (57 ± 3) J mol^−1^ K^−1^; Ala–Ala: Δ*c*^SL^_p0Ala–Ala_ (*T*^G^_0Ala–Ala_) = (84 ± 18) J mol^−1^ K^−1^ and Δ*c*^SL^_p0Ala–Ala_ (*T*^SL^_0Ala–Ala_) = (62 ± 18) J mol^−1^ K^−1^. The solid squares depict specific heat capacity of solid Gly–Gly from literature.^60^ In order to avoid crystallization on cooling, higher scanning rates are required and might be able to achieved with ultra-fast scanning nanocalorimetry.^61,62^

Fig. 4 shows the glass transition step (solid line) in specific heat capacity for Gly–Gly, Gly–Ala, Ala–Gly and Ala–Ala. The heat capacity of solid phase, *c*^S^_p0i_, measured with conventional DSC, as well as the heat capacity of liquid phase, *c*^L^_p0i_, determined from the glass transition step were linearly fitted in order to extrapolate to melting temperature. The heat capacity difference between liquid and solid phase were determined at glass transition temperature, Δ*c*^SL^_p0i_ (*T*^G^_0i_) and at melting temperature, Δ*c*^SL^_p0i_ (*T*^SL^_0i_). It should be mentioned that we assumed the heat capacity of glass and crystal state equal to each other. This assumption is reasonable, while the heat capacity difference between glass and crystal phases often lower than the uncertainty of heat capacity determination with FSC technique (approx. 10%).^54–59^

Unfortunately, no heat capacity difference for cyclo(Ala–Gly) can be determined as cyclo(Ala–Gly) crystallizes on cooling from melted state, even at cooling rate 20 000 K s^−1^.

The experimental melting temperatures, melting enthalpy, and heat capacity differences at glass transition temperature and at melting temperature of dipeptides, as well as the melting properties implemented in PC-SAFT, are listed in Table 4. The melting properties of Ala–Gly differs considerably from that of cyclo(Ala–Gly). This implies that Ala–Gly does not undergo cyclization into cyclo(Ala–Gly) upon heating in FSC conditions and allows determining the melting properties of dipeptides without accounting for this chemical process. So, the big difference in the melting properties justifies the observed difference in the solubility data of Ala–Gly and cyclo(Ala–Gly).

**Table 4:** Experimental melting properties (*T*^SL^_0i_, Δ*h*^SL^_0i_ and Δ*c*^SL^_p0i_) of the dipeptides used in this work

	FSC				
	*M* [g mol^−1^]	*T* ^SL^_0i_ [K]	Δ*h*^SL^_0i_ [kJ mol^−1^]	Δ*c*^SL^_p0i_ (*T*^G^_0i_) [J mol^−1^ K^−1^]	Δ*c*^SL^_p0i_ (*T*^SL^_0i_) [J mol^−1^ K^−1^]
Gly–Gly	132.12	593 ± 7	40 ± 6	84 ± 6	51 ± 6
Gly–Ala	146.15	551 ± 7	41 ± 5	91 ± 6	55 ± 6
Ala–Gly	146.15	611 ± 7	52 ± 7	82 ± 3	57 ± 3
Ala–Ala	160.17	606 ± 7	45 ± 7	84 ± 18	62 ± 18
Cyclo(Ala–Gly)	128.13	526 ± 7	24 ± 4	—	—

### Solubility of dipeptides

**Fig. 5:**
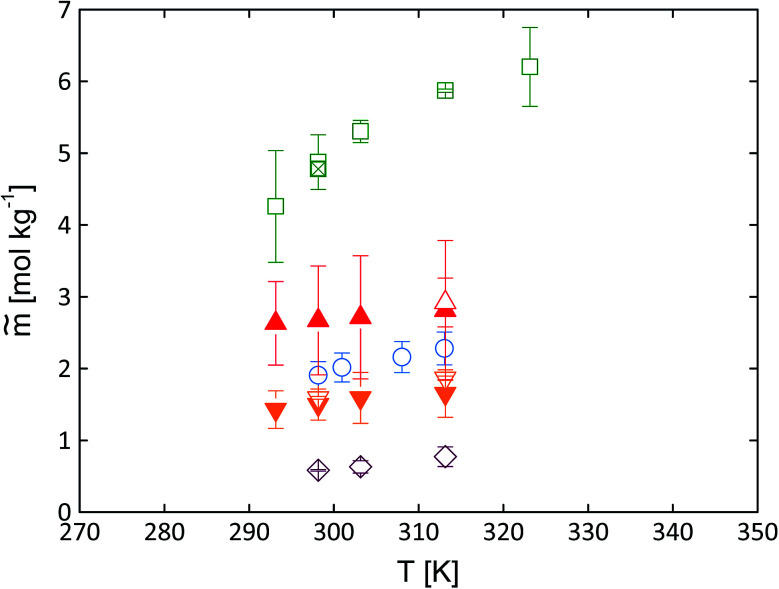
Experimental dipeptide solubility at pH = 7 in water as molality *vs.* temperature. Full symbols present data measured by photometric method; empty symbols by gravimetric method. Gly–Ala (squares + “*x*”-filled square^38^); Ala–Ala (up-triangles); Gly–Gly (circles);^11^ Ala–Gly (down-triangles) and cyclo(Ala–Gly) (diamonds). The experimentally determined values are given in Tables S5 and S6 (pH 7) and Tables S7 and S8 (pH at saturated solutions) in the ESI.†

The dipeptide solubility was adjusted to pH = 7 using eqn (3) and (4). For these equations, p*K*_a_ values of the dipeptides and pH values of the saturated solutions are required. The p*K*_a_ values were determined using the platform Chemicalize®. The results of the pH measurements of the aqueous unbuffered saturated dipeptide solutions are given in Table S2 in the ESI.† The solubility results are illustrated in Fig. 5.

As expected, the solubility of all dipeptides increases with increasing temperature. Some literature works have already reported solubility data of dipeptides in water. The temperature-dependent solubility of Gly–Gly as well as Gly–Ala in water are listed in the literature ref. 11 and 38, respectively. These solubility data were repeated in the present work accounting also for pH correction using eqn (3) and (4). In addition to the already existing data, the temperature-dependent solubility of Gly–Ala in water was determined gravimetrically in the current work. Overall, Fig. 5 illustrates that the data agree well with each other.

### PXRD results

To prove that the crystals form in the equilibrium with saturated aqueous solution in the solubility are equal to those studied by FSC the PXRD analysis was carried out. This is one of the requirements for application of eqn (1) in its presented form. The results of the PXRD measurements are shown in Fig. S6 in the ESI.† The PXRD measurements should determine if the crystal structure of the pure substance changes with the solvent water. A change in the crystal structure would be determined in a shift of the intensities to different angles. As shown in Fig. S6 in the ESI† the crystal structures of the pure dipeptides are the same as in equilibrium with saturated water solution. No change in crystal structures could be detected during the measurements.

### Expected solubility behavior

Eqn (1) shows that solubility depends on melting properties and solvent–solute interactions. From a chemical perspective, it is known that an increase in hydrophobicity of the peptides will decrease their solubility in water based on hydrophobic alkyl chain residue. The amino acids glycine (Gly), l-alanine (Ala), l-valine (Val) and l-leucine (Leu) differ only in the length of the alkyl side chain. Comparing the solubility behavior of these amino acids in aqueous solutions^63^ it can be stated that the increasing alkyl residual size leads to decreasing solubility in water. By comparing solubilities of dipeptides to those of Gly and Ala, it would have been expected that

(1) The solubility of the dipeptides are lower than of the respective amino-acids constituents,

(2) The solubility of the isomeric dipeptides Gly–Ala and Ala–Gly is expected to be equal and

(3) the solubility of Gly–Ala and of Ala–Gly is assumed to be in between the solubilities of Gly and Ala and

(4) The solubility is expected to decrease with the increase in alkyl chain length as in the case of amino acids. That, Gly–Gly is expected to have a larger solubility than Gly–Ala and larger than Ala–Gly, which in turn are expected to have a higher solubility than Ala–Ala.

**Fig. 6:**
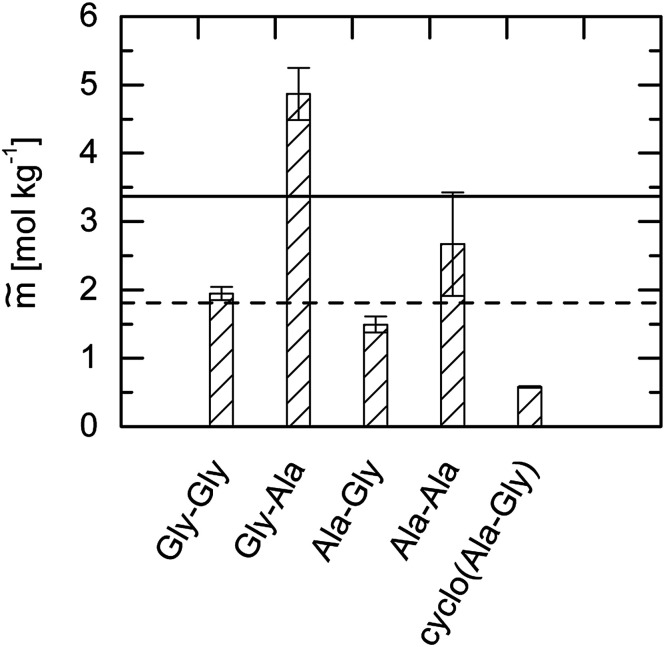
Solubility in water. Bars: mean values of the photometric and gravimetric determined dipeptide solubility data at *T* = 298.15 K and pH = 7. Lines represent the corresponding amino-acid solubility at *T* = 298.15 K and pH = 7: solid line: Gly^24^ and dashed line: Ala.^24^

Unexpectedly, none of these expected behaviors were found experimentally. The comparison of the solubilities of the four dipeptides compared to the amino acids Gly and Ala is illustrated in Fig. 6.

First, it becomes clear from Fig. 6 that amino acids are not necessarily more soluble in water than dipeptides. In the following, only those dipeptides are considered that possess the amino acid Gly in the first place of the primary structure. As soon as another Gly with a dipeptide binding is placed in the second place (Gly–Gly), the solubility decreases. However, if an Ala is present at second place (Gly–Ala), the solubility is increased and even exceeds the solubility of the glycine.

Now, we consider peptides that possess the amino acid Ala at the first place of the primary structure. Combining this with Gly at the second place (Ala–Gly) causes a strong decrease in the water solubility that it is even lower than the solubility of the Ala. However, as soon as Ala occurs at second place again (Ala–Ala), the solubility is increased compared to the amino acid Ala.

As observed the order of the sequence has an important role for the observed solubility data. In total as shown in Fig. 8 the dipeptides solubility follows the orderGly–Ala > Gly > Ala–Ala > Gly–Gly > Ala > Ala–Gly > cyclo(Ala–Gly)

**Fig. 7:**
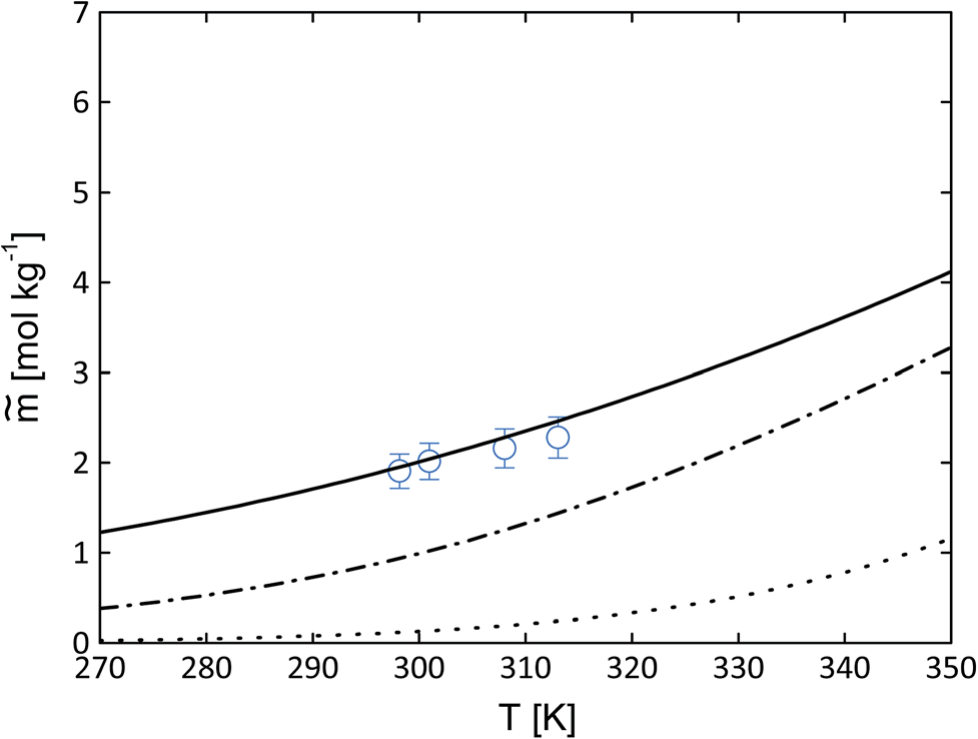
Influence of the difference of the heat capacities on the solubility behaviour for Gly–Gly (circles^11^) in water as molality *vs.* temperature. Lines represent PC-SAFT predictions with the parameters from Table 3 and FSC-measured melting properties from this work (Table 4). Dotted line: eqn (1) with Δ*c*^SL^_p0i_ = 0, dotted-dashed line: eqn (1) with Δ*c*^SL^_p0i_ = const. = 51 J mol^−1^ K^−1^, solid line: eqn (1) with eqn (2).

**Fig. 8:**
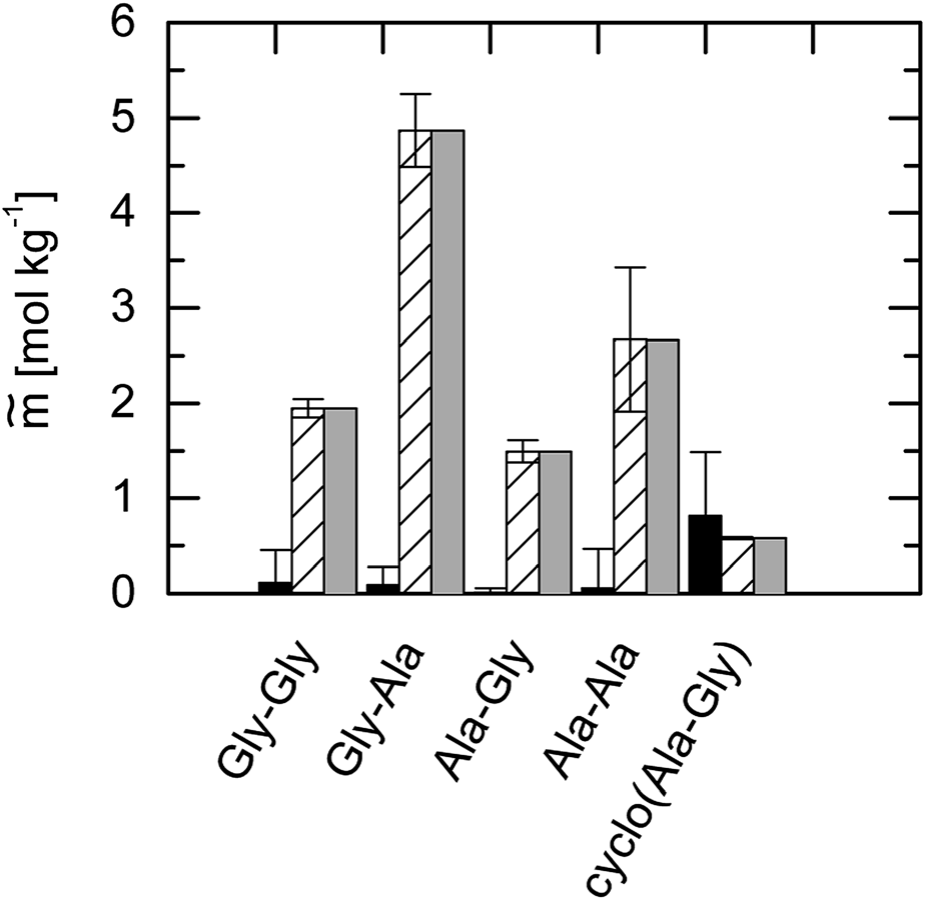
Influence of the activity coefficient on the solubility in molality in water expressed as difference between ideal and experimental solubility. Black (Ideal): calculated solubility at *T* = 298.15 K from eqn (1) based on the FSC-measured melting properties in experimental uncertainty assuming an ideal mixture *γ* = 1. Shaded (Exp.): mean value of the photometric and gravimetric determined solubility data at *T* = 298.15 K and pH = 7. Grey (PC-SAFT): calculated solubility at *T* = 298.15 K from eqn (1) based on the PC-SAFT used melting properties from Table 4 including the activity coefficient.

Besides the chemical structure and the kind of polymorphic solid form that has been produced upon dissolution, the dipeptide-water interactions (activity coefficient) and the melting properties of the solid dipeptide (*T*^SL^_0i_, Δ*h*^SL^_0i_ and Δ*c*^SL^_p0i_) determine the exact values of solubility according to eqn (1). This is discussed in the following.

### Influence of melting properties on solubility behavior

According to eqn (1) the melting properties of the pure dipeptide have a strong influence on solubility. The experimental melting properties determined in this work are listed in Table 4. The Δ*c*^SL^_p0i_ values are given at glass transition and at melting temperature. Note, that the latter value is a temperature-extrapolated value using temperature-dependent *c*^S^_p0i_ and *c*^L^_p0i_ values of the pure dipeptides. From here onwards, the difference of heat capacities at melting temperature Δ*c*^SL^_p0i_ (*T*^SL^_0i_) was used for the solubility prediction using eqn (1).

The solubility data at pH = 7 between Ala–Gly and cyclo(Ala–Gly) are compared with each other. Due to different melting properties as mentioned in advance as well as different solubility, a thermally induced cyclization can be excluded. It is assumed that no cyclization has been occurred for all the other dipeptides.

The solubility of the isomeric dipeptides Gly–Ala and Ala–Gly are very different (Fig. 5). In contrast, the activity coefficients of Gly–Ala and Ala–Gly in water are the same within experimental uncertainties (Fig. S2 in the ESI†). Thus, a difference in the melting properties is the only explanation for the observed solubility between Ala–Gly and Gly–Ala are the different crystal structures which yield to different melting properties.

The general effect of the melting properties on solubility modeling using eqn (1) is discussed briefly in the following. Decreasing melting temperature *T*^SL^_0i_, melting enthalpy Δ*h*^SL^_0i_ as well as increasing heat capacity difference of Δ*c*^SL^_p0i_ lead to higher solubility values. Based on the experimental data the melting temperature of dipeptides appears to be about *T*^SL^_0dipeptides_ ≈ 600 K (except for Gly–Ala with *T*^SL^_0Gly−Ala_ = 551 + −7 K), while Δ^SL^_p0dipeptides_ ≈ 55 J mol^−1^ K^−1^. Thus, it can be concluded that the main reason for the solubility difference is the enthalpy of melting which is different for Ala–Gly and Gly–Ala (Δ*h*^SL^_0Ala−Gly_ = (52 ± 7) kJ mol^−1^ and Δ*h*^SL^_0Gly−Ala_ = (41 ± 5) kJ mol^−1^). The lower the value for the melting enthalpy the higher the solubility according to eqn (1) given that activity coefficients play a minor role. As we found that the activity coefficients of Ala–Gly and Gly–Ala in water are equal, the difference in the melting enthalpy mainly explains the higher solubility of Gly–Ala over Ala–Gly.

It can be concluded from the experimental melting properties that the uncertainty of *T*^SL^_0i_ and of Δ*c*^SL^_p0i_ measured with FSC is sufficiently high. Changing *T*^SL^_0i_ and of Δ*c*^SL^_p0i_ within their uncertainty values only slightly influences the solubility according to eqn (1). In contrast, changing the FSC-measured melting enthalpy within its error bars strongly influences solubility according to eqn (1).^24^

### Influence of heat capacity on solubility behavior

According to eqn (1) the calculation on the solubility can be performed with different approaches to the heat capacity difference Δ*c*^SL^_p0i_. It can be either equal to zero, constant over the temperature or temperature-dependent. The importance to take in account the difference of the heat capacities is illustrated in Fig. 7.

Fig. 7 shows the influence of Δ*c*^SL^_p0i_ on the solubility predictions using the melting properties of Gly–Gly (see Table 5, PC-SAFT). The dotted line represents eqn (1) with the approach of Δ*c*^SL^_p0i_ = 0. The solubility prediction is lower than the experimentally determined aqueous solubility of Gly–Gly. The dashed-dotted line represents the assumption of Δ*c*^SL^_p0i_ = const. = 51 J mol^−1^ K^−1^. The solubility increases compared to Δ*c*^SL^_p0i_ = 0. The solid line takes in account the temperature dependency of Δ*c*^SL^_p0i_ as described in eqn (2). The solubility increases again and is in good agreement to the experimental data of Gly–Gly in a broad temperature range. Based on the heat capacity results (Fig. 4) the Δ*c*^SL^_p0i_ is neither equal to zero nor constant over the temperature range. Thus, all the PC-SAFT calculations in this work have been done with the approach of the linear temperature-dependent Δ*c*^SL^_p0i_(*T*) expressed by eqn (2). The individually slope and interceptions of the heat capacity of the liquid and solid phase are listed in Table 5.

**Table 5:** Melting properties used for PC-SAFT solubility predictions in this work

	PC-SAFT					
	*T* ^SL^_0i_ [K]	Δ*h*^SL^_0i_ [kJ mol^−1^]	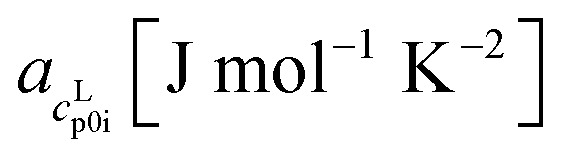	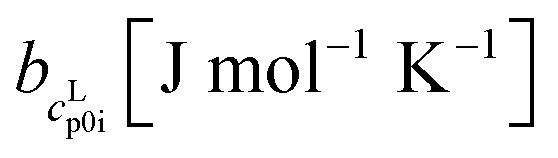	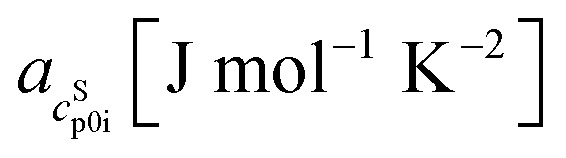	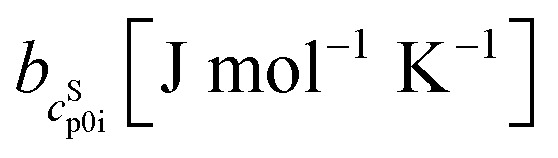
Gly–Gly	593	42.02	0.256	185.660	0.415	41.175
Gly–Ala	551	38.04	0.253	219.937	0.474	44.123
Ala–Gly	611	45.01	0.357	169.339	0.475	41.054
Ala–Ala	606	41.72	0.396	175.923	0.503	49.918
Cyclo(Ala–Gly)	533	22.69	—	—	—	—

### Influence of activity coefficients on solubility behavior

The solubility of dipeptides depends on the kind of medium in which the dipeptides were dissolved. This is expressed in the activity coefficient of the dipeptide at saturation. In this work the activity coefficients of the dipeptides Gly–Gly, Gly–Ala, Ala–Gly, Ala–Ala and cyclo(Ala–Gly) in water were predicted with PC-SAFT. This allowed predicting solubility by combining the activity coefficients with the melting properties from FSC using eqn (1). “Prediction” means that all of the PC-SAFT parameters were fitted to solubility-independent data such as activity coefficients and mixture densities in water. The predicted values agree with the experimental data within the experimental-determined error range (Fig. 8).

**Fig. 9:**
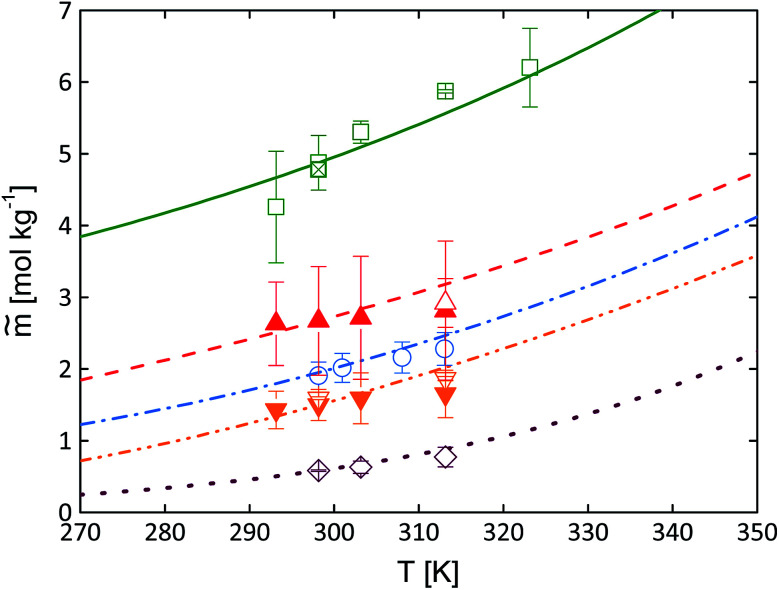
Dipeptides solubility at pH = 7 in water as molality *vs.* temperature. Symbols represent experimental data. Solid symbols present measurements using photometric method; open symbols present measurements using gravimetric method. Gly–Ala (squares + "x" filled square^38^); Ala–Ala (up-triangles); Gly–Gly (circles^11^); Ala–Gly (down-triangles); cyclo(Ala–Gly) (diamonds). Lines represent PC-SAFT predictions with the parameters from Table 3 and FSC-measured melting properties from this work. Gly–Ala (solid line), Ala–Ala (dashed line), Gly–Gly (dashed-dotted line), Ala–Gly (dashed-double dotted line) and cyclo(Ala–Gly) (dotted line). The experimental determined values are given in Tables S5 and S6 (pH = 7) and Tables S7 and S8† (pH at saturated solutions).

Fig. 8 shows the solubility (in molality units) for ideal and experimental conditions. First, the ideal (=ideal mixture) solubility was calculated by setting activity coefficient equal to one at *T* = 298.15 K using eqn (1). The error bars of this calculation are based on using the FSC-measured melting properties within their uncertainty limits. It can be seen that the calculation of solubility assuming ideal mixture leads to very small solubility values. Thus, assuming ideal solution does not allow matching the experimentally determined solubility data. Therefore, the dipeptide-water interaction was accounted for by means of PC-SAFT. The success of the predictions shown in Fig. 8 mean that it is crucially important to take the interactions between dipeptide and water into account in order to predict solubility successfully. Note, that the FSC-measured melting properties were modified within their experimental uncertainty (see Table 4) by adjusting them to the experimental data shown in Fig. 8, and the melting properties are listed in Table 5. In the following, these were used to predict the experimental solubility data as shown in Fig. 9. It can be observed that PC-SAFT allows for quantitative prediction of the experimental solubility behavior. The used melting properties agree very well with the FSC-determined melting properties, and the methods cross-validate the experimental melting properties as well as the accuracy of activity-coefficient predictions and the experimental solubility data.

In sum, it can be concluded the FSC-measured melting properties as well as the PC-SAFT refined melting properties do not deviate much, which can be considered as an excellent result. It could be shown that both the FSC-measured melting data and the use of a thermodynamic model for the solvent–solute interactions are indispensable for the correct temperature-dependent solubility prediction of dipeptides.

## Conclusion

In a previous work^24^ we have shown that melting properties *T*^SL^_0i_ and Δ*h*^SL^_0i_ of amino acids can be determined experimentally by means of FSC. In this work, the method was applied to dipeptides, and the *T*^SL^_0i_ and *Dh*^SL^_0i_, as well as heat capacity difference Δ*c*^SL^_p0i_(*T*) were determined directly by using FSC for the five dipeptides Gly–Gly, Gly–Ala, Ala–Gly, Ala–Ala and cyclo(Ala–Gly). First of all, different melting properties as well as solubility data have been determined for cyclo(Ala–Gly) and Ala–Gly, indicating that thermally-induced cyclization does not occur during the determination of melting properties of Ala–Gly. This excludes errors caused by chemical transformation. In a next step, solubility of the dipeptides in water was measured experimentally *vs.* temperature. A change of crystal structure during the solubility measurements was excluded by PXRD measurement verifications. The difference in the experimentally-observed solubility of Gly–Gly, Gly–Ala, Ala–Gly and Ala–Ala was found to be correlated to the melting properties of the dipeptides.

Finally, PC-SAFT parameters were fitted to solubility-independent thermodynamic properties (activity coefficients, osmotic coefficients and mixture densities). Based on these parameters and the FSC-determined melting properties (*T*^SL^_0i_, Δ*h*^SL^_0i_ and a linear temperature-dependent Δ*c*^SL^_p0i_(*T*)) the solubility of the dipeptides in water was predicted with PC-SAFT. The predicted solubility was found to be in very good agreement with the experimental determined solubility data. This cross-validates PC-SAFT as method to quantitatively predict activity coefficients at saturation as well as FSC to accurately measure the melting properties of compounds that usually decompose before melting upon measuring in conventional DSC apparatuses. The availability of our new experimental melting properties will improve also other predictive models in the future up to high temperatures.

## Conflicts of interest

There are no conflicts to declare.

## Supplementary Material

RA-009-C9RA05730G-s001
