# Supplementary material for: Melting properties of peptides and their solubility in water. Part 1: dipeptides based on glycine or alanine
Source: RSC Adv. 2019 Oct 14;9(56):32722–34. doi: 10.1039/c9ra05730g (PMC9073158; doi:10.1039/c9ra05730g)
Supplement: RA-009-C9RA05730G-s001 [file RA-009-C9RA05730G-s001.pdf]

Electronic Supplementary Material (ESI) for RSC Advances.
This journal is © The Royal Society of Chemistry 2019

Melting Properties of Peptides and their Solubility in Water. Part I: Dipeptides based on Glycine or Alanine

Hoang Tam Do[#a], Yeong Zen Chua[#b,c*], Jonas Habicht[a], Marcel Klinksiek[a], Moritz Hallermann[a], Dzmitry Zaitsau[c,d], Christoph Schick[b,c,e], Christoph Held[a*]

[a] *Laboratory of Thermodynamics, Department of Biochemical and Chemical Engineering, Technische Universität Dortmund, Emil-Figge-Str. 70, 44227 Dortmund, Germany*

[b] *Institute of Physics, University of Rostock, Albert-Einstein-Str. 23-24, 18051 Rostock, Germany*

[c] *Competence Centre CALOR, Faculty of Interdisciplinary Research, University of Rostock, Albert-Einstein-Str. 25, 18051 Rostock, Germany*

[d] *Institute of Chemistry, University of Rostock, Dr-Lorenz-Weg 2, 18051 Rostock, Germany*

[e] *Chemical Institute A.M. Butlerov, Kazan Federal University, 18 Kremlyovskaya Street, Kazan 420008, Russian Federation*

[#] *shared first authors*

[*] *E-mail: christoph.held@tu-dortmund.de (solubility), yeong.chua@uni-rostock.de (FSC)*

Supporting Information

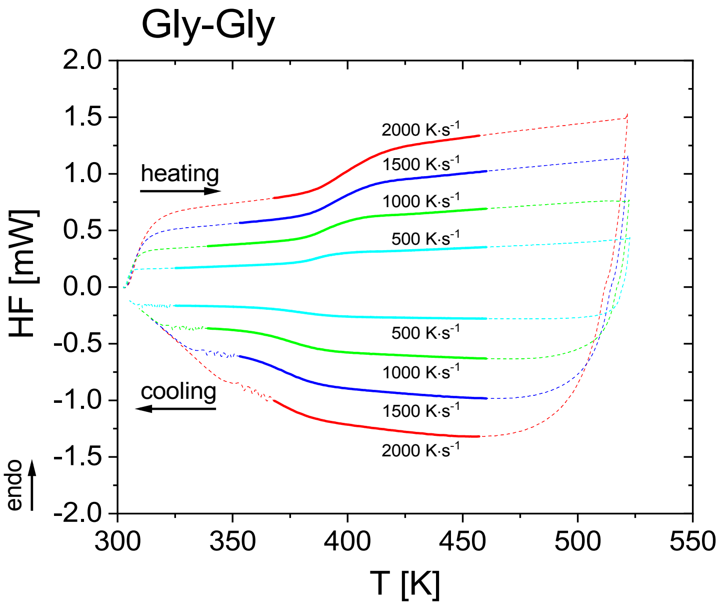

Figure S1 *Heating step #8 and cooling step #9 for glassy Gly-Gly. The dashed lines represented the actual heating/cooling curves, while thick solid lines represented the mutual accessible temperature range in both heating and cooling steps. The scanning rates used were 2000 K·s^{-1} (most outer heating/cooling cycle), 1500 K·s^{-1}, 1000 K·s^{-1} and 500 K·s^{-1} (most inner heating/cooling cycle). The accessible temperature range increases with decreasing scanning rate.*

Table S1 *pK_a values for the dipeptides used in this work from the platform Chemicalize®.*

Dipeptide	$pK_{a,1}$	$pK_{a,2}$	$pK_{a,3}$
Gly-Gly	3.52	8.14	15.32
Gly-Ala	3.61	8.14	13.82
Ala-Gly	3.66	8.39	14.32
Ala-Ala	3.73	8.39	13.82
cyclo(Ala-Gly)	10.94	12.17	-

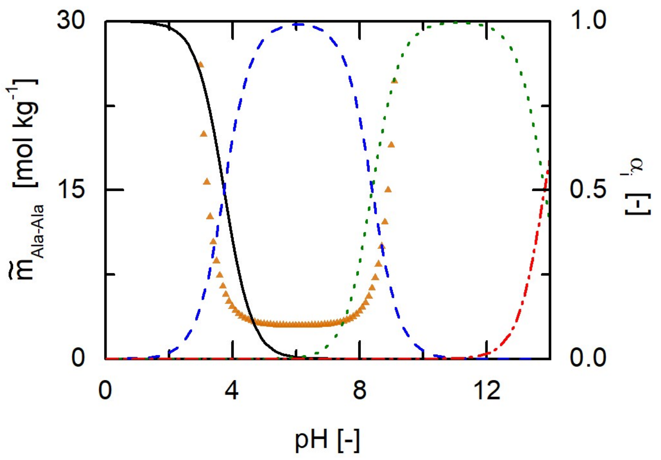

Figure S2 *Species distribution of Ala-Ala and the converted solubility (triangles) at T = 298.15 K and p = 1 bar. Lines represent the different species. positive net charged specie (solid), zero net charge specie (dashed), negative net charged specie (dotted), two negative charged species (dashed-dotted).*

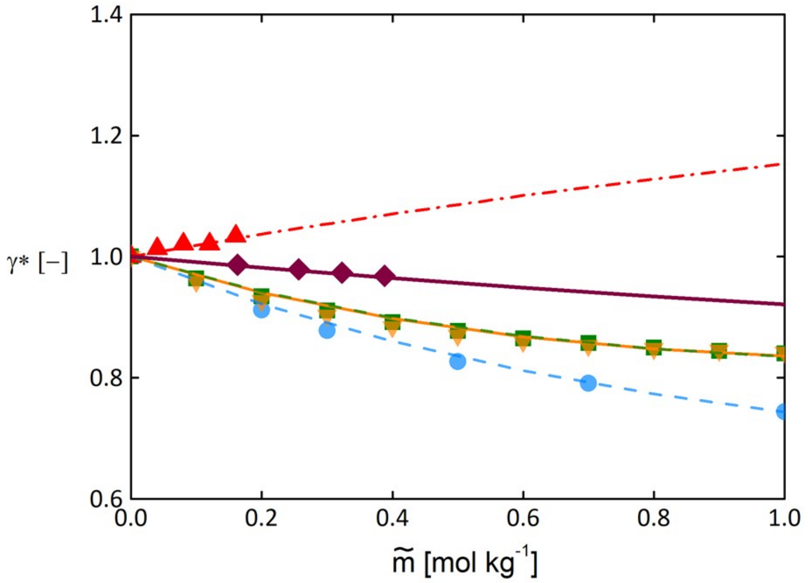

Figure S3 *Activity coefficients of Gly-Gly[11], Gly-Ala[11], Ala-Gly[11], Ala-Ala[7] and cyclo(Ala-Gly) + water solutions at 298.15 K. Solid symbols are experimental data: Gly-Gly (circles); Gly-Ala and Ala-Gly (squares and down-triangles); Ala-Ala (up-triangles) and cyclo(Ala-Gly) (diamonds). The lines are the respective PC-SAFT modeling results with parameters from Table 3.*

Table S2 *Activity coefficients of dipeptides in aqueous solutions at different temperatures.*

**Gly-Gly**		**Gly-Ala**		**Ala-Gly**		**Ala-Ala**		**cyclo(Ala-Gly)**	
$\tilde{m}$ [mol kg^{-1}]	γ [-]	$\tilde{m}$ [mol kg^{-1}]	γ [-]	$\tilde{m}$ [mol kg^{-1}]	γ [-]	$\tilde{m}$ [mol kg^{-1}]	γ [-]	$\tilde{m}$ [mol kg^{-1}]	γ [-]
0.2	0.912	0.1	0.964	0.1	0.959	0.1	0.980	0.16	0.987
0.3	0.878	0.2	0.934	0.2	0.927	0.2	0.97	0.26	0.979
0.5	0.827	0.3	0.911	0.3	0.903	0.3	0.967	0.32	0.974
0.7	0.791	0.4	0.892	0.4	0.884	0.4	0.968	0.39	0.969
1.0	0.744	0.5	0.877	0.5	0.870	0.5	0.972	-	-
1.2	0.723	0.6	0.865	0.6	0.860	0.6	0.978	-	-
1.5	0.696	0.7	0.857	0.7	0.853	0.7	0.984	-	-
1.7	0.685	0.8	0.85	0.8	0.848	0.8	0.991	-	-
-	-	0.9	0.844	0.9	0.845	0.9	0.999	-	-
-	-	1.0	0.84	1.0	0.842	1.0	1.011	-	-

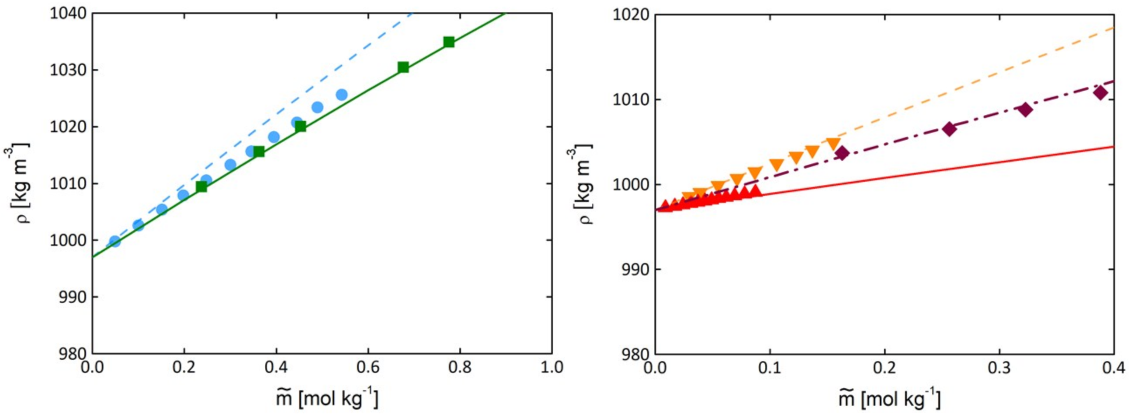

Figure S4 *Densities of aqueous dipeptide solutions vs. molality. Solid symbols represent experimental data, Lines represent PC-SAFT modeling results with the parameters from Table 3. LEFT: Gly-Gly (circles[38]) and Gly-Ala (squares[38]). RIGHT: Ala-Gly (down-triangles[11]), Ala-Ala (up-triangles[11]) and cyclo(Ala-Gly) (diamonds).*

Table S3 Density of dipeptides in aqueous solutions at 298.15 K.

**Gly-Gly**		**Gly-Ala**		**Ala-Gly**		**Ala-Ala**		**Cyclo(Ala-Gly)**	
$\tilde{m}$ [mol kg^{-1}]	ρ [kg m^{-3}]	$\tilde{m}$ [mol kg^{-1}]	ρ [kg m^{-3}]	$\tilde{m}$ [mol kg^{-1}]	ρ [kg m^{-3}]	$\tilde{m}$ [mol kg^{-1}]	ρ [kg m^{-3}]	$\tilde{m}$ [mol kg^{-1}]	ρ [kg m^{-3}]
0.05	999.78	0.24	1009.39	0.16	1004.946	0.17	1004.97	0.16	1003.7
0.10	1002.59	0.36	1015.62	0.14	1004.028	0.23	1008.21	0.26	1006.5
0.15	1005.37	0.45	1020.02	0.12	1003.32	0.32	1012.28	0.32	1008.8
0.20	1007.87	0.68	1030.45	0.11	1002.465	0.46	1018.62	0.39	1010.8
0.25	1010.52	0.78	1034.86	0.09	1001.503	0.66	1026.87	-	-
0.30	1013.27	0.97	1043.58	0.07	1000.715	0.81	1032.83	-	-
0.35	1015.64	1.03	1045.91	0.05	999.873	0.94	1038.21	-	-
0.39	1018.16	1.37	1059.24	0.04	999.068	0.17	1004.97	-	-
0.45	1020.72	1.56	1066.36	0.03	998.574	0.23	1008.21	-	-
0.49	1023.4	-	-	-	-	0.32	1012.28	-	-
0.54	1025.61	-	-	-	-	-	-	-	-

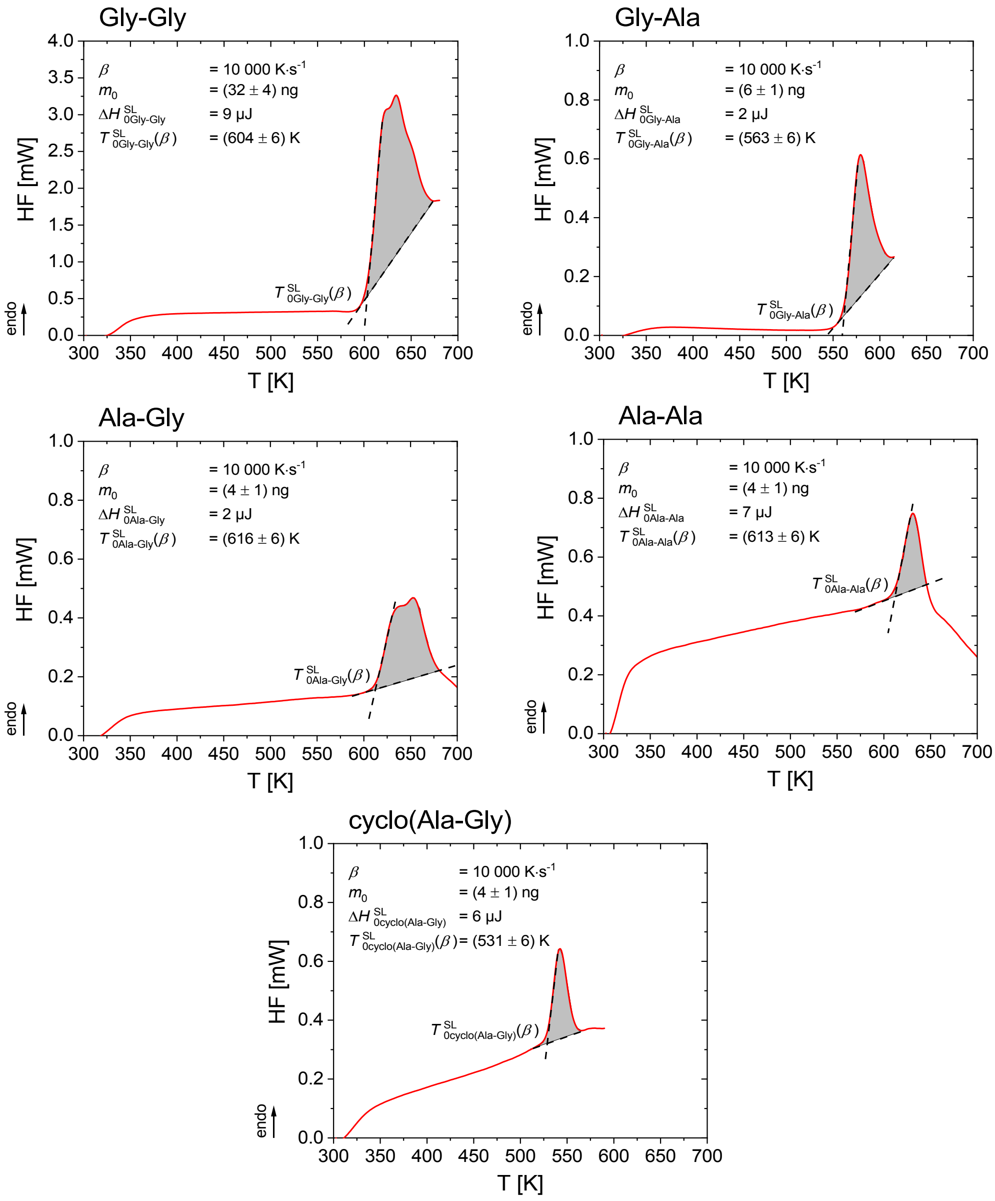

Figure S5 *Heat flow curve of Gly-Gly, Gly-Ala, Ala-Gly, Ala-Ala and cyclo(Ala-Gly) in heating step #5. The melting temperature, $T_{0i}^{SL}(\beta)$, is determined as the onset of the melting peak, while the enthalpy, ΔH_{0i}^{SL}, as area under curve.*

Table S4 *pH values of the unbuffered saturated dipeptide – water solutions at different temperatures*

Dipeptide \ T [K]	*293.15*	*298.15*	*301.15*	*308.15*	*313.15*
Gly-Gly	5.90 ± 0.01	5.85 ± 0.01	5.86 ± 0.01	5.85 ± 0.02	5.82 ± 0.01
Dipeptide \ T [K]	*293.15*	*298.15*	*303.15*	*313.15*	*323.15*
Gly-Ala	6.17 ± 0.01	6.08 ± 0.02	6.09 ± 0.01	5.96 ± 0.05	5.88 ± 0.03
Ala-Gly	5.95 ± 0.01	5.85 ± 0.01	5.85 ± 0.01	5.80 ± 0.04	5.60 ± 0.02
Ala-Ala	5.78 ± 0.02	5.77 ± 0.02	5.79 ± 0.01	5.71 ± 0.01	5.65 ± 0.01
cyclo(Ala-Gly)	-	6.84 ± 0.11	6.77 ± 0.20	6.73 ± 0.09	-

Table S5 *Solubility of dipeptide in molality in aqueous solutions at pH 7 at different temperatures (gravimetric method)*

Dipeptide \ T [K]		*298.15*	*301.15*	*308.15*	*313.15*
Gly-Gly	-	1.91 ± 0.19	2.01 ± 0.20	2.16 ± 0.21	2.28 ± 0.22
Dipeptide \ T [K]	*293.15*	*298.15*	*303.15*	*313.15*	*323.15*
Gly-Ala	3.95 ± 1.13	4.94 ± 0.31	5.26 ± 0.16	6.03 ± 0.27	6.20 ± 0.58
Ala-Gly	-	1.59 ± 0.02	-	-	1.87 ± 0.03
Ala-Ala	-	-	-	-	3.06 ± 0.34
cyclo(Ala-Gly)	-	0.58 ± 0.01	0.63 ± 0.09	0.77 ± 0.14	-

Table S6 *Solubility of dipeptide in molality in aqueous solutions at pH 7 at different temperatures (photometric method)*

Dipeptide \ T [K]	*293.15*	*298.15*	*303.15*	*313.15*	*323.15*
Ala-Gly	1.43 ± 0.26	1.49 ± 0.22	1.59 ± 0.36	1.65 ± 0.33	-
Ala-Ala	2.63 ± 0.58	2.67 ± 0.76	2.71 ± 0.86	2.81 ± 0.97	-

Table S7 *Solubility of dipeptide in molality in aqueous unbuffered solutions at different temperatures (gravimetric method)*

Dipeptide \ T [K]		*298.15*	*301.15*	*308.15*	*313.15*
Gly-Gly		1.79 ± 0.20	1.89 ± 0.21	2.03 ± 0.58	2.14 ± 0.22
Dipeptide \ T [K]	*293.15*	*298.15*	*303.15*	*313.15*	*323.15*
Gly-Ala	3.72 ± 1.06	4.64 ± 0.29	4.94 ± 0.15	5.64 ± 0.26	5.80 ± 0.55
Ala-Gly	-	1.54 ± 0.02	-	-	1.81 ± 0.03
Ala-Ala	-	-	-	-	2.97 ± 0.34
cyclo(Ala-Gly)	-	0.58 ± 0.01	0.63 ± 0.09	0.77 ± 0.14	-

Table S8 *Solubility of dipeptide in molality in aqueous solutions at pH 7 at different temperatures (photometric method)*

Dipeptide \ T [K]	*293.15*	*298.15*	*303.15*	*313.15*	*323.15*
Ala-Gly	1.38 ± 0.26	1.45 ± 0.22	1.53 ± 0.36	1.59 ± 0.33	-
Ala-Ala	2.55 ± 0.58	2.59 ± 0.76	2.63 ± 0.86	2.73 ± 0.97	-

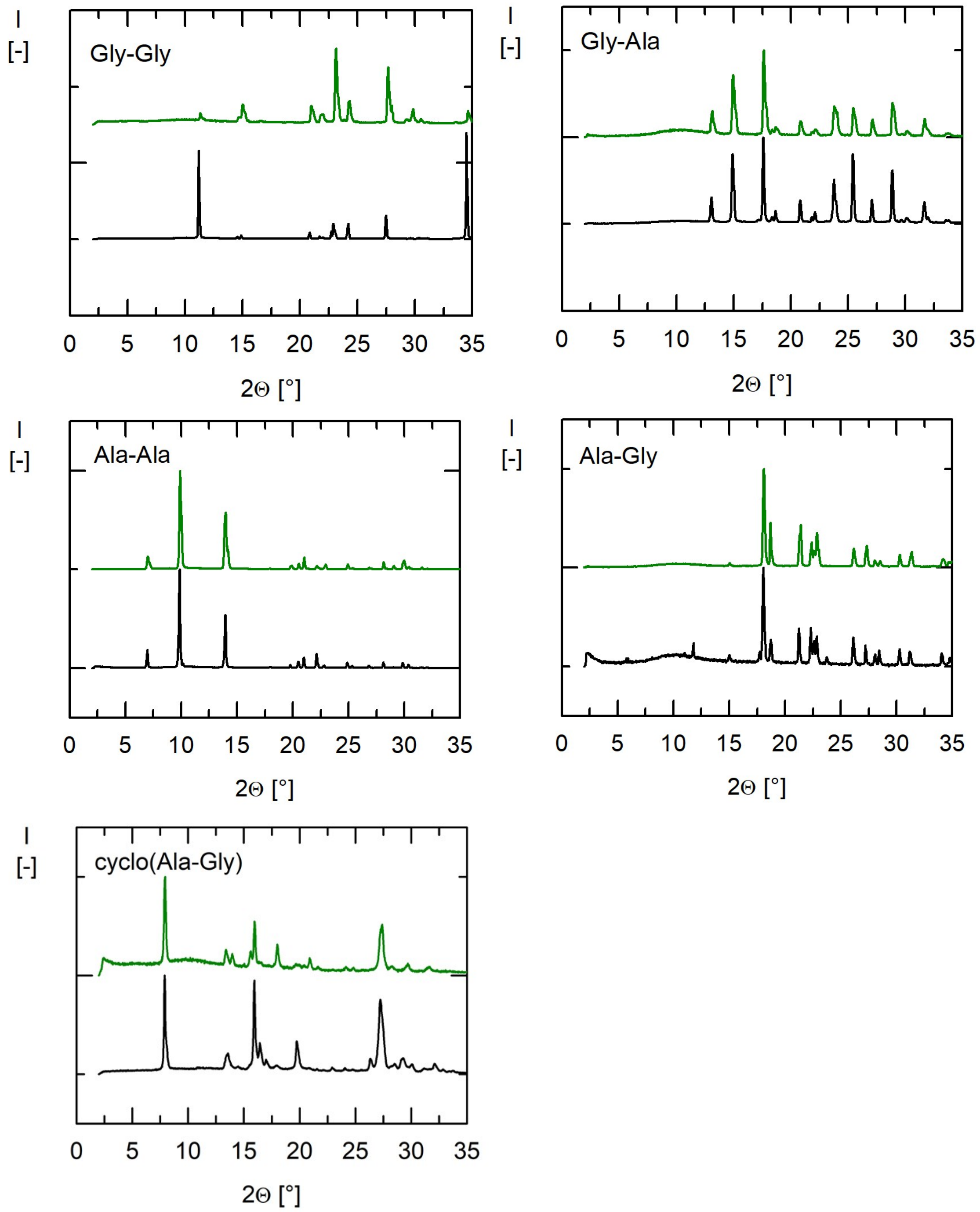

Figure S6 *Diffractogram of Gly-Gly, Gly-Ala, Ala-Ala, Ala-Gly and cyclo(Ala-Gly) as intensity vs. angle 2Θ. The upper line represents the pure substance from the supplier without further treatment. The lower line represents the substance in equilibrium state with unbuffered water.*
